# AKT Axis, miR-21, and RECK Play Pivotal Roles in Dihydroartemisinin Killing Malignant Glioma Cells

**DOI:** 10.3390/ijms18020350

**Published:** 2017-02-10

**Authors:** Ying-Ying Shao, Tao-Lan Zhang, Lan-Xiang Wu, He-Cun Zou, Shuang Li, Jin Huang, Hong-Hao Zhou

**Affiliations:** 1 Institute of Life Sciences, Chongqing Medical University, 1 Yixueyuan Road, Yuzhong District, Chongqing 400016, China; yingying_shao1226@163.com (Y.-Y.S.); lxwu2008@126.com (L.-X.W.); zouhecun@outlook.com (H.-C.Z.); 2 Department of Clinical Pharmacology, Xiangya Hospital, Central South University, Changsha 410008, China; CSU_ZTL@163.com (T.-L.Z.); m15200923235@163.com (S.L.); huangjin879288@163.com (J.H.); 3 Hunan Key Laboratory of Pharmacogenetics, Institute of Clinical Pharmacology, Central South University, Changsha 410078, China

**Keywords:** dihydroartemisinin (DHA), glioma, miR-21, RECK, apoptosis, metastasis, invasion

## Abstract

Dihydroartemisinin (DHA), a semi-synthetic derivative of artemisinin, is known to play important roles in inhibiting proliferation rate, inducing apoptosis, as well as hindering the metastasis and invasion of glioma cells, but the underlying mechanisms are still unclear so far. In this study, methyl thiazolyl tetrazolium (MTT), colony-forming, wound healing, invasion, and apoptosis assays were performed to investigate the effect of DHA on malignant glioma cells. Results showed that DHA induced apoptosis of malignant glioma cells through Protein Kinase B (AKT) axis, induced death of malignant glioma cells by downregulating miR-21, and inhibited the invasion of malignant glioma cells corresponding with up-regulation of the reversion-inducing-cysteine-rich protein with kazal motifs (RECK). These results revealed that AKT axis, miR-21, and RECK play pivotal roles in DHA killing malignant glioma cells, suggesting that DHA is a potential agent for treating glioma.

## 1. Introduction

Malignant glioma is the most common primary central nervous system tumor, accounting for 80% of all brain and central nervous system tumors [1]. Despite aggressive surgical resection followed by radiation and chemotherapy, the median survival time of patients with glioblastoma multiforme (World Health Organization grade IV) is only 12–14 months [2]. Its aggressiveness and critical health care matter mainly arise from the extensive proliferation and insidious invasion capabilities of malignant glioma cells [3]. To date, the search for novel and more efficient chemo-agents against this deadly disease has become a major research focus in the field of glioblastoma multiforme treatment.

Dihydroartemisinin (DHA), which is one of the main active metabolites of arteminisin (Qinghaosu), is used as anti-malarial agents [4,5]. Previous studies have reported that DHA has strong anticancer activities through inhibiting the proliferation and invasion of different types of cancers such as gastric cancer [6], hepatocellular carcinoma [7], breast cancer [8], ovarian cancer [9], and lung cancer in vitro [10] and in vivo [11]. In glioma cells, DHA has also been shown to inhibit proliferation, metastasis, and invasion, and enhance radio sensitivity, but these molecular mechanisms need to be further investigated [12,13,14].

The Protein Kinase B (AKT) axis (PI3K/AKT) is one of the most vital pathways affecting cell proliferation and apoptosis [15,16,17]. It has been known that aberrant AKT activation (phosphorylated Ser/Thr residues in AKT) is closely related with the abnormal survival and proliferation of various cancer cells [16]. Moreover, p53, a tumor suppressor gene, can induce apoptosis through suppressing Bcl-2 as well as activating Bax [18,19]. Previous studies have shown that the AKT axis (PI3K/AKT) is involved in the proliferation and apoptosis of glioma cells [20], but the role of this axis (AKT/p53/Bcl-2/Bax) in DHA treatment is still unknown.

MicroRNAs (miRNAs), 19- to 25-nucleotide, are endogenous, small, non-coding RNAs and regulate gene expression by targeting the 3′-untranslated region (3′-UTR) [21]. Emerging evidence has indicated that miRNAs are involved in the anti-cancer effects of many reagents [22]. MiR-21 is an oncogenic miRNA and is over-expressed in malignant glioma [23,24]. Previous studies revealed that down-regulated miR-21 caused chemical-induced cancer cell death [25,26]. Additionally, in recent years, there has been much experimental evidence that the reversion-inducing-cysteine-rich protein with kazal motifs (RECK) could reduce the metastasis and invasion of various cancer cells, including malignant glioma [27,28], through regulating matrix metalloproteinases (MMPs) [29] and associated proteins such as Cadherin families [30]. However, there is no report focusing on the roles of miR-21 and RECK in the treatment of malignant glioma cells with DHA.

Therefore, the aim of this study was to optimize the therapeutic effect of DHA against glioma cells and to study the roles of AKT axis (AKT/p53/ Bcl-2/Bax), miR-21, and RECK in the anti-cancer activity induced by DHA. In the current study, we studied the molecular mechanisms for the anticancer activities of DHA in glioma cells and implicated miR-21 and RECK as a target for therapeutic intervention in DHA-induced cancer cell death and invasion.

## 2. Results

### 2.1. DHA Inhibits the Growth of Human Malignant Glioma Cells In Vitro

**Figure 1:**
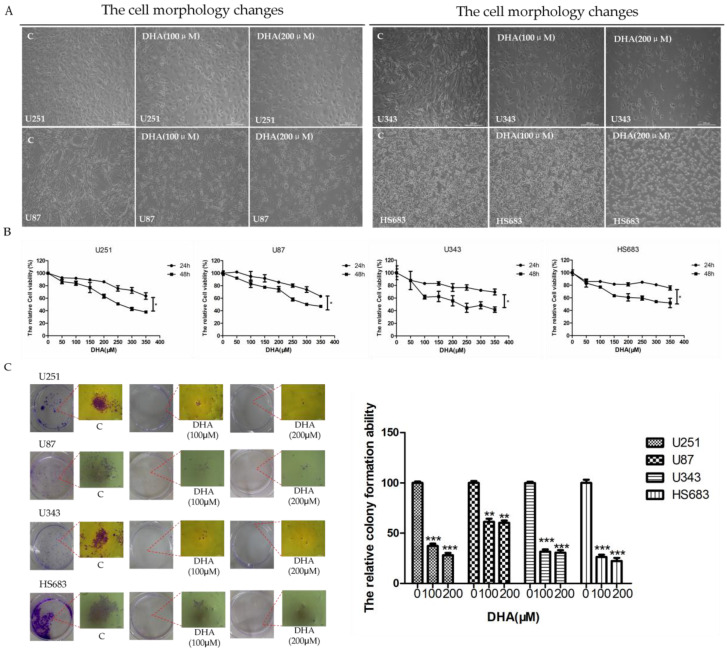
Inhibitive effect of dihydroartemisinin (DHA) on the growth of human malignant glioma cells in vitro. (**A**) Malignant glioma cells (U251, U87, U343, and HS683) were treated with 100 μM and 200 μM DHA for 48 h. Cell morphological changes were determined under phase contrast microscope at 10×; (**B**) Malignant glioma cells (U251, U87, U343, and HS683) were treated with a series of DHA concentrations (0–350 μM) for 24 or 48 h followed by methyl thiazolyl tetrazolium (MTT) assay. Values were mean ± SD (*n* = 3). * *p* < 0.05 as compared with negative control cells; (**C**) Foci formation of U87, U251, U343, and HS683 cells was determined. After indicated treatments, malignant glioma cells were trypsinized and plated in duplicates at low density. After 10 days, formed colonies were stained with crystal violet. Values were mean ± SD (*n* = 3), ** *p* < 0.001, *** *p* < 0.0001 as compared with negative control cells.

To study the inhibitive effect of DHA on malignant glioma cells growth, cell morphology detection, methyl thiazolyl tetrazolium (MTT) assay, and colony forming ability test were performed in a series of cell lines including U251, U87, U343, and HS683. We first evaluated the growth inhibitory effects of DHA on human malignant glioma cell lines using MTT assays. DHA reduced malignant glioma cells viability in a dose- and time-dependent manner (Figure 1B). Compared to cells treated with 0.1% dimethyl sulfoxide (DMSO, negative control group), a dose-dependent reduction in the viability of the U251 cells was observed that ranged from 8% to 37% after 24 h, and 14% to 63% after 48 h of treatment with DHA, as shown in Figure 1B. Under identical conditions, similar effects of DHA were observed on treatment of U87, U343, and HS683 cells (Figure 1B). Furthermore, the effect of DHA-induced reduction in the viability of U251 cells was comparatively higher than that observed for the other cell lines after 48 h of treatment with DHA. After treatment with 100 and 200 μM DHA for 48 h, significant apoptotic morphological changes (such as smaller, round, and blunt in size) of malignant glioma cells (U251, U87, U343, and HS683) were observed in a dose dependent manner compared to control groups (Figure 1A). Moreover, DHA could also suppress colony formation, demonstrating that DHA not only inhibited long-term survival of malignant glioma cells, but also damaged the pseudopodia, which played a pivotal role in the metastasis and invasion of malignant glioma cells (Figure 1C). In summary, these results demonstrated that DHA could inhibit the growth of human malignant glioma cells in vitro through inducing cell apoptosis and death, and, moreover, that DHA might also inhibit the metastasis and invasion of malignant glioma cells.

### 2.2. AKT Axis Plays a Pivotal Role in DHA-Induced Malignant Glioma Cells Apoptosis

**Figure 2:**
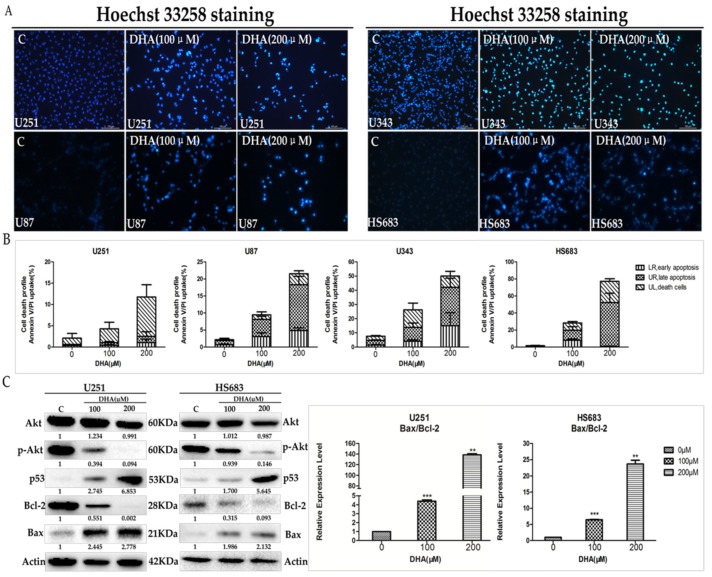
Effect of DHA on malignant glioma cells apoptosis by regulating AKT axis. The indicated cells were treated with 100 and 200 μM DHA for 48 h. (**A**) Cells stained with Hoechst 33258 were detected and calculated by fluorescent photomicrographs at 10×; (**B**) cells were labeled with Annexin V/Propidium Iodide (AnnexinV/PI) and detected by flow cytometry. Values were mean ± SD (*n* = 3); (**C**) the proteins associated with AKT/p53/Bcl-2/Bax axis in malignant glioma cells were determined by western blot analysis. The changes of Bax/Bcl-2 ratio were evaluated by western blot analysis. Values were mean ± SD (*n* = 3). ** *p* < 0.001, *** *p* < 0.0001 as compared with negative control cells.

To further investigate the effect of DHA on the apoptosis of malignant glioma cells, Hoechst 33258 staining and Annexin V/Propidium Iodide (AnnexinV/PI) flow-cytometry assay were applied to measure cell apoptosis after 48 h of treatment with DHA. Hoechst 33258 staining showed that DHA caused condensed and/or fragmented nuclei in malignant glioma cells (Figure 2A). Figure 2B showed that DHA increased apoptosis of malignant glioma cells in a dose-dependent manner. When the concentration of DHA reached 200 μM, the apoptosis rate was significantly higher than that of untreated cells. According to previous studies, various signaling pathways have been reported to account for DHA-induced apoptosis in different cancer cells. Of these proposed mechanisms, the ability of DHA to affect the AKT axis was especially noteworthy [31,32,33], so we investigated its role in DHA inducing malignant glioma cell apoptosis in this study. Western blot analysis showed that DHA inhibited AKT phosphorylation, activated p53, and increased the ratio of Bax/Bcl-2 in a dose-dependent manner (Figure 2C). Taken together, these results suggested that AKT/p53/Bcl-2/Bax axis played a pivotal role in DHA-induced malignant glioma cells apoptosis.

### 2.3. MiR-21 Modulation and Its Effect on DHA-Induced Malignant Glioma Cells Death

**Figure 3:**
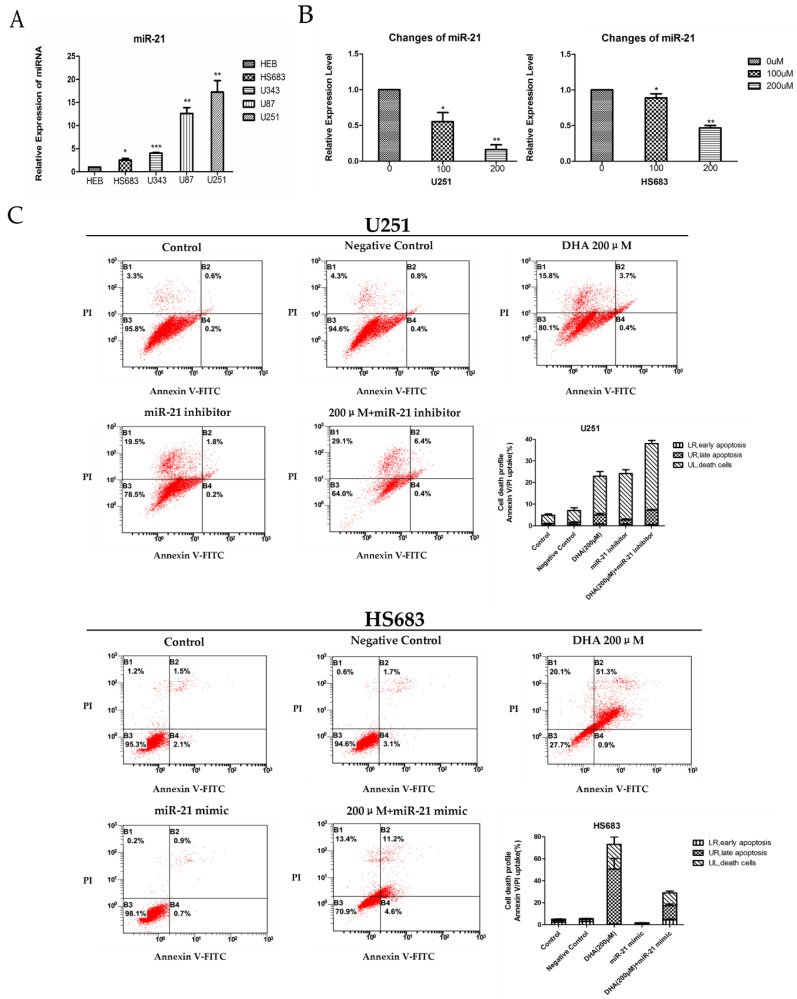
Effect of DHA on malignant glioma cells death by downregulating miR-21. (**A**) Relative expression of miR-21 in normal human glial cell line (HEB) and malignant glioma cells. Values were mean ± SD (*n* = 3), * *p* < 0.05, ** *p* < 0.01, *** *p* < 0.001 as compared with negative control cells; (**B**) DHA downregulated miR-21 in malignant glioma cells (U251 and HS683). Values were mean ± SD (*n* = 3), * *p* < 0.05, ** *p* < 0.01 as compared with negative control cells; (**C**) malignant glioma cells (U251 and HS683) with different treatments assayed by AnnexinV/PI flow cytometry. Right lower and upper quadrant and left upper quadrant showed cell deaths. Values were mean ± SD (*n* = 3).

Previous studies have indicated that miR-21 is an anti-apoptosis factor in glioblastoma cells, and miR-21 knock-down increases apoptosis [34,35,36]. Thus, a series of experiments were performed to further investigate whether modulation of miR-21 affected DHA-induced malignant glioma cell death. As shown in Figure 3A, miR-21 was highly over-expressed in malignant glioma cells in comparison to the normal human glial cell line (HEB). After 48 h of treatment with DHA in malignant glioma cells (U251 and HS683), DHA decreased miR-21 expression in a dose-dependent manner (Figure 3B). Furthermore, Annexin V/Propidium Iodide (AnnexinV/PI) flow-cytometry assay showed that miR-21 inhibitor could intensify DHA-induced U251 cell death, and miR-21 mimic could partly reverse DHA-induced HS683 cells death (Figure 3C). These data suggested that miR-21 played an important role in DHA-induced malignant glioma cell death.

### 2.4. The Up-Regulation of RECK Plays a Pivotal Role in the Inhibitive Effect of DHA on Metastasis and Invasion of Malignant Glioma Cells

**Figure 4:**
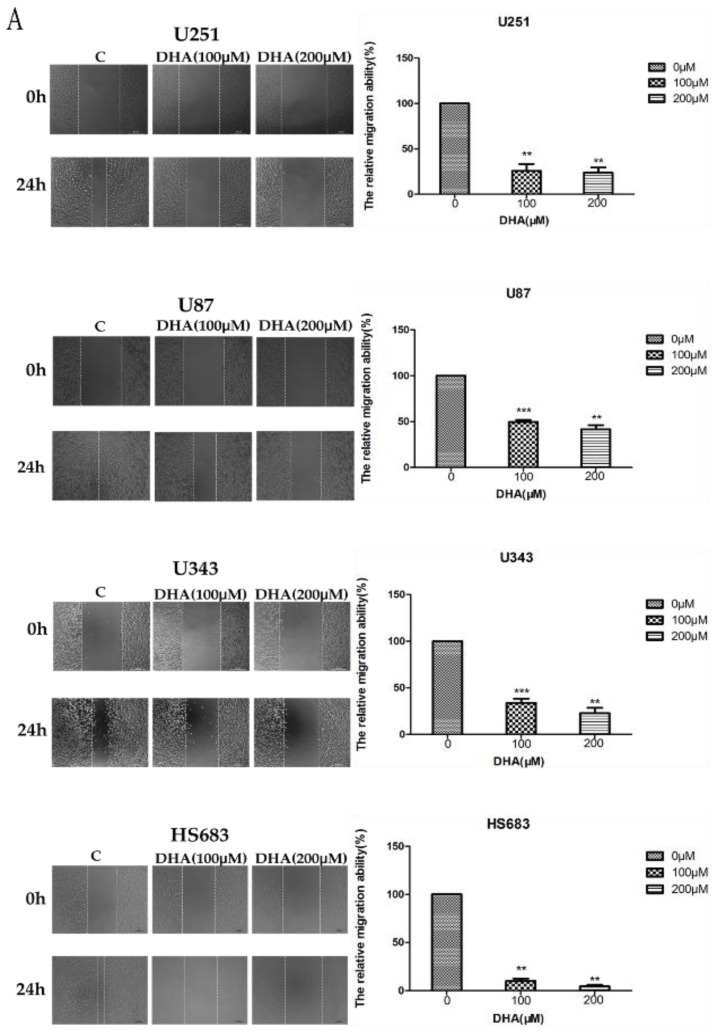

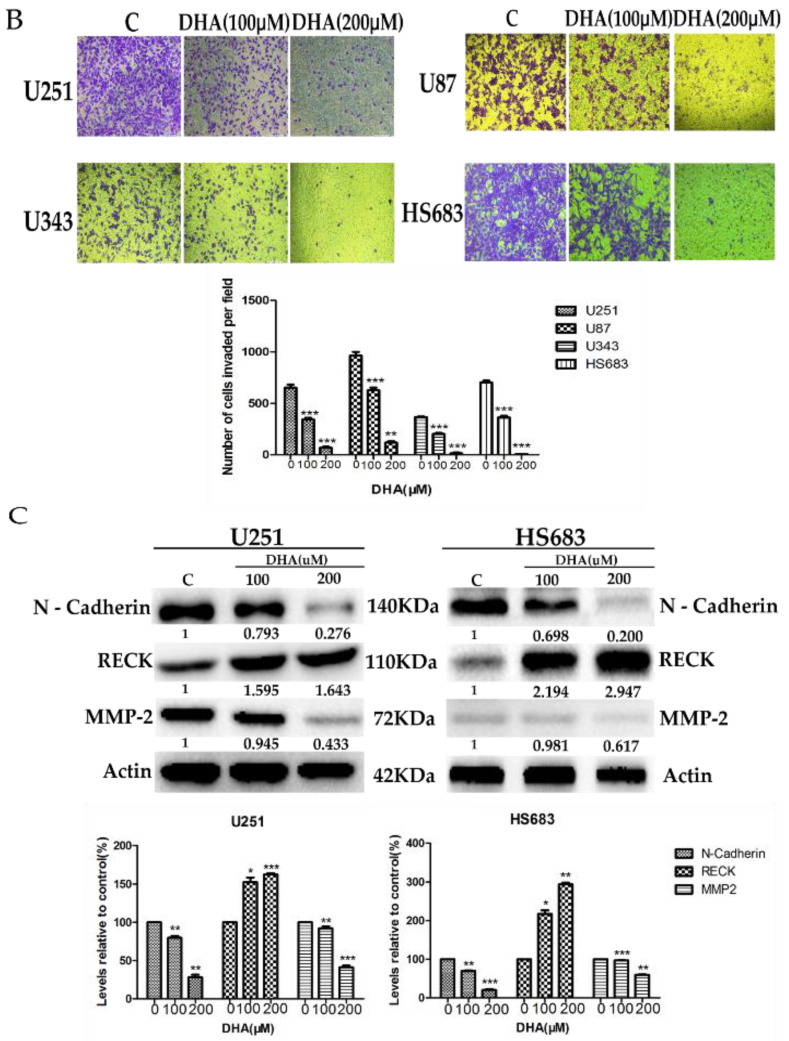
Inhibitive effect of DHA on the metastasis and invasion of malignant glioma cells by upregulating RECK (reversion-inducing-cysteine-rich protein with kazal motifs). (**A**) The inhibitive effects of DHA on malignant glioma cells migratory ability. Values were mean ± SD (*n* = 3), ** *p* < 0.01, *** *p* < 0.001 as compared with negative control cells; (**B**) the effects of DHA on malignant glioma cells invasion ability. Values were mean ± SD (*n* = 3), ** *p* < 0.01, *** *p* < 0.001 as compared with negative control cells; (**C**) the changes of proteins associated with metastasis and invasion induced by DHA. Malignant glioma cells were treated with DHA for 24 h, then the protein levels of RECK, MMP-2, and *N*-Cadherin were assessed by western blot analysis. Values were mean ± SD (*n* = 3), * *p* < 0.05, ** *p* < 0.01, *** *p* < 0.001 as compared with negative control cells.

To further explore the signal mechanism on the inhibitive effect of DHA on metastasis and invasion of malignant glioma cells, we performed wound healing, transwell chambers, and western blot analysis assays as described in the “Materials and Methods” section. To confirm that the inhibitive effect of DHA on metastasis and invasion of glioma cells was a direct effect on the cell’s capacity for metastasis and invasion as opposed to a reduced cell viability or cell death, we treated glioma cells with 100 and 200 μM DHA for 24 h; in this condition, DHA had no significant effect on cell death or cell viability from our previous MTT results. In the wound healing assay, vehicle-treated malignant glioma cells covered at least 70% of the gap at 24 h. In contrast, the scratch was still largely uncovered at 24 h in DHA-treated malignant glioma cells (Figure 4A). These results indicated that DHA suppressed the metastasis of malignant glioma cells. As shown in Figure 4B, in comparison with untreated control cells, DHA significantly reduced the invasion of malignant glioma cells through matrigel membrane in a dose-dependent manner. Western blot analysis showed that DHA could upregulate the protein level of RECK as well as downregulate the marker proteins (*N*-Cadherin and MMP-2) for migration and invasion ability of cancer cells in malignant glioma cells in a dose-dependent manner (Figure 4C). These results indicated that the up-regulation of RECK played a pivotal role in the inhibitive effect of DHA on metastasis and invasion of malignant glioma cells.

### 2.5. Relationship of RECK Expression with Clinicopathological Features of Glioma Patients

**Figure 5:**
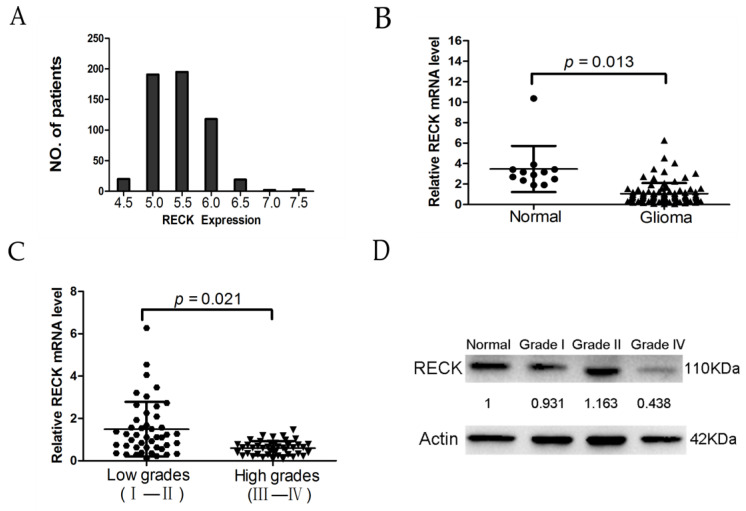
Relative expression of RECK in glioma tissues. (**A**) Analysis of RECK expression levels in Glioblastoma multiforme (GBM) tumor tissues from the Cancer Genome Atlas (TCGA) data. The histograms showed distribution of the RECK expression levels in 548 GBM tumor tissues; (**B**) qRT-PCR analysis of RECK expression level in 12 normal brain tissues and 88 glioma tissues; (**C**) RECK mRNA levels in lower grades glioma tissues and higher grades glioma tissues; (**D**) the protein level of RECK was assessed by western blot analysis in normal brain tissues and glioma tissues.

We further investigated the expressional profile of RECK in glioblastoma multiforme (GBM) tumor tissues. As shown in Figure 5A, the Cancer Genome Atlas (TCGA) data of RECK mRNA levels in 548 GBM tumor tissues assessed by Affymetrix U133A arrays revealed that RECK mRNA levels ranged from 4.06 to 7.25 with more than 95% samples below median expression level 6.0, so the expression of RECK mRNA in the majority of the GBM tumor tissues was low. Furthermore, we validated the RECK mRNA expression by real-time quantitative reverse transcription-PCR (qPCR), and our results showed that the RECK mRNA level was significantly lower in malignant gliomas compared to lower grade gliomas and non-tumor brain tissues (Figure 5B,C). Moreover, western blot analysis showed the RECK protein level was similar with regards to its mRNA in malignant gliomas, lower grade gliomas, and non-tumor brain tissues (Figure 5D).

To further elucidate the significance of RECK in glioma, we calculated the correlation of RECK expression with clinicopathological features of 88 glioma patients (as shown in Table 1) and found that highly expressed RECK was closely related to tumor grade of glioma (*p* = 0.021). However, no association of RECK expression with age, gender, and tumor location (*p* = 0.498, 0.655, and 0.104, respectively) was detected. Thus, we speculated that RECK might have important implications for the progression of glioma, and these data implied that DHA was a potential therapeutic treatment of glioma.

## 3. Discussion

**Figure 6:**
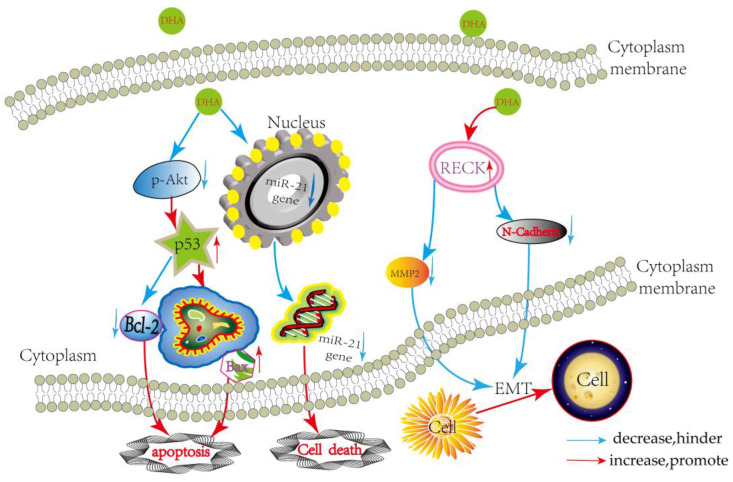
Schematic representation of the signaling pathway mediating DHA lethality to malignant glioma cells.

Malignant glioma is the most common primary brain tumor in adults. Current standard therapy is mainly dependent on surgical treatment, followed by radiotherapy and adjuvant temozolomide. Despite the advances in chemotherapy for glioblastoma, the prognosis for patients is poor with a five-year survival rate of only 9%. To our knowledge, the activity of chemotherapeutic agents is limited, with a very low response rate because of drug resistance and other reasons, thus more favorable chemotherapeutic agents are urgently needed. DHA, a metabolite of artemisinin, can inhibit proliferation, induce apoptosis, and suppress metastasis and invasion ability of various cancers. In glioma cells, DHA has also been shown to inhibit proliferation, metastasis, and invasion and enhance radio sensitivity, but these molecular mechanisms need to be further investigated [12,13,14]. In the present study, our data suggest that DHA shows antiproliferative and proapoptotic activities through regulating AKT/p53/Bcl-2/Bax axis, and downregulating miR-21. We also assessed the effects of DHA on the migrating and invasive capabilities of malignant glioma cells; the results demonstrate that DHA can inhibit the metastasis and invasion ability of malignant glioma cells in a dose- and time-dependent manner mainly via activating RECK (Figure 6). These results suggest that DHA is a potential therapeutic treatment of glioma.

Many chemotherapeautic agents serve as a killer of cancer cells through the apoptosis pathway. As an important anti-apoptotic signaling pathway, the PI3K/AKT signaling cascade has attracted considerable attention. Accumulative evidence has shown that the PI3K/AKT pathway plays an important role in the transduction of growth factor signals, which regulate gene expression to control cell survival and proliferation [15,16,17]. In malignant glioma cells, Du et al. indicated that DHA suppressed the Raf/MEK/ERK and PI3K/AKT pathways in BT325 and C6 cell lines [20]. Moreover, the activation of pro-apoptotic proteins p53, Bax and the inhibition of anti-apoptotic protein Bcl-2 were involved in the apoptosis process of various cancer cells in previous studies [18], but this effect of DHA on glioma cells has not been investigated. In our present study, DHA decreased p-Akt levels drastically and it also increased p53 and the ratio of Bax/Bcl-2, which induces the apoptosis of human malignant glioma cells in a concentration- and time-dependent manner. However, as reported in Figure 2B, U251 cells died mainly through necrosis rather than apoptosis, while in the other three cell lines, apoptosis was the major cause of cell death. The underlying mechanism should be further elucidated in future studies.

Recent studies have indicated that miRNAs play critical roles in the process of chemicals against cancers [22], and many studies have indicated that miR-21 is over-expressed in GBM cells and functions as an oncogene in GBM cells [23,24,25,26]. Sun et al. demonstrated that the modulation of miR-15b and miR-16 mediated the apoptosis effect of DHA for gastric cancer cells [5]. However, whether miR-21 plays a pivotal role in DHA against malignant glioma or not remained unclear in previous studies. In our study, DHA treatment significantly decreased the expression of onco-gene miR-21 in U251 and in HS683 in a dose-dependent manner (Figure 3B), and the changes of miR-21 expression could influence the effect of DHA on the death of U251 and HS683 (Figure 3C). These results demonstrate that miR-21 plays a pivotal role in DHA against malignant glioma. However, how DHA decreases the expression of miR-21 in glioma cells remains unclear and this mechanism remains largely unknown. In all, our current results firstly suggest that DHA induces malignant glioma cell death by downregulating miR-21 and that the changes of miR-21 expression influence the effect of DHA on the death of malignant glioma cells.

As indicated in the results section, DHA could damage malignant glioma cells pseudopodia; we speculated that it could inhibit the metastasis and invasion of malignant glioma cells, but the potential mechanism was still unclear. Previous studies have reported that RECK is newly found as a transformation-suppressor gene, which can inhibit the process of tumor metastasis and invasion through regulating the expression of MMPs and Cadherin families [27,28,29,30]. Thus, we further investigated whether DHA inhibits the metastasis and invasion of malignant glioma cells by upregulating RECK. In this study, RECK is activated by DHA treatment in glioma cells (Figure 4E), corresponding with the observed inhibition of migratory and invasive ability. In addition, the protein levels of MMP-2 and *N*-Cadherin were reduced in glioma cells treated with DHA. Consequently, we firstly demonstrated that DHA suppresses the metastasis and invasion of glioma cells via up-regulating RECK in glioma cells, implying that DHA is a candidate agent to treat the metastasis and invasion of glioma in the first clinical trials. However, RECK is a validated target of miR-21 [27], whether there is a relationship between the effect of DHA on the up-regulation of RECK and the down-regulation of miR-21 still remains to be elucidated.

## 4. Materials and Methods

### 4.1. Human Tissue Samples

Eighty-eight patients with grade I to IV gliomas that underwent surgical resection in Hunan Cancer Hospital (Changsha, China) between 2007 and 2013 were enrolled in the Institute of Clinical Pharmacology review board-approved study (The ethical permission code is CTXY-1300041-3, the permission date is 13 September 2013 and the name of the ethics committee is Institute of Clinical Pharmacology, Hunan Key Laboratory of Pharmacogenetics, Central South University, Changsha, China). Twelve normal brain samples (from patients with brain injuries) were collected for controls. The tissue samples were flash frozen in liquid nitrogen immediately after resection and stored at −80 °C until further processing.

### 4.2. Cell Culture and Reagents

Malignant glioma U87, U251 cell lines were kindly provided by Wu Sihan (Department of Pharmacology, Zhongshan School of Medicine, Sun Yat-sen University, Guangzhou, China). Malignant glioma U343 and HS683 cell lines were kindly provided by Gong Zhicheng (Department of Pharmacy, Xiangya Hospital, Central South University, Changsha, China). All cell lines were cultured in Dulbecco’s Modified Eagle Medium (DMEM) with 10% fetal bovine serum (BI) at 37 °C in an incubator with 5% CO_2_. DHA and dimethyl sulfoxide (DMSO) were purchased from Sigma-Aldrich (St. Louis, MO, USA). DHA was dissolved in DMSO. The maximum concentration of DMSO in media was 0.1% (*v*/*v*). Cells treated with DMSO only served as a vehicle control.

### 4.3. Cell Proliferation Assay

Cells were plated in 96-well plates at a density of 5 × 10^3^ and treated with various concentrations of DHA or 0.1% DMSO (negative control) for 24 and 48 h. The effects of DHA on U87, U251, U343, HS683 cell proliferation were evaluated by methyl thiazolyl tetrazolium (MTT) assay (Sigma-Aldrich) according to the manufacturer’s instruction. The viability properties of glioma cells did not vary after treatment with the solvent (only) used to dissolve DHA.

### 4.4. Colony Formation Assay

To assay the effects of DHA on colony formation of glioma cells, 400 viable malignant glioma cells (U87, U251, U343, and HS683) were seeded in six-well plates in triplicate for 24 h, then continually maintained in complete medium with or without a series doses of DHA for 10 days. Foci were fixed with 4% polyoxymethylene and stained with 0.1% crystal violet (Beyotime, C0121, Shanghai, China). The stained foci were washed by Phosphate Buffered Saline (PBS) three times and then detected by microscope. The foci were further dissolved in 500 μL DMSO and quantified by spectrophotometer at 540 nm.

### 4.5. Apoptosis Assays

Apoptosis was evaluated by the apoptotic morphology, Hoechst 33258 Staining, flow cytometry. Apoptotic morphology was observed by microscope (Nikon, Japan) and Hoechst 33258 Staining (Beyotime, Beijing, China). Cells with condensed and fragmented nuclei were considered and calculated as the apoptotic cells. For flow cytometry analysis, cells were collected after treating with DHA for 48 h and stained with Annexin V-FITC Apoptosis Detection Kit (BD Biosciences, San Jose, CA, USA) according to the manufacturer’s instruction and analyzed by a flow cytometer (Beckman Coulter, Brea, CA, USA).

### 4.6. Wound Healing Assay

Malignant glioma cells (U87, U251, U343, and HS683) were cultured in six-well plates. After cells approached almost 100% confluence, the supernatant was absorbed and the cells were scraped with the fine end of 20 µL pipette tips. Then the cells were washed with PBS to remove detached cells and medium was added with series doses of DHA. Cell metastasis was photographed by microscope at 0 and 24 h, respectively. Remodeling was measured as diminishing distance across the induced injury, normalized to the 0 h control, and expressed as outgrowth. The migratory properties of glioma cells did not vary after treatment with the solvent (only) used to dissolve DHA.

### 4.7. Matrigel Invasion Assay

For the matrigel invasion assay of glioma cells (U87, U251, U343, and HS683), filters were precoated with 60 μL Matrigel (Corning, Corelle, NY, USA) for 3 h at 37 °C. The two chambers were separated with Matrigel-coated Millipore membranes (6.5 mm diameter filters, 8.0 μM pore size) 3 h later. Culture medium containing 10% FBS (600 μL) was added to lower chambers, cells (1 × 10^4^ cells/100 μL serum-free medium) were placed in the upper chamber of the corning chambers and DHA was added in combination to the upper chamber (200 μL). After the assay had been run for 24 h at 37 °C, non-invaded cells were removed from the upper surface of the filter. Cells on the lower surface of the membrane were fixed with 4% polyoxymethylene and stained with crystal violet (Beyotime, Beijing, China). Cell numbers were counted under an optical microscope. The invasion properties of glioma cells did not vary after treatment with the solvent (only) used to dissolve DHA. Each experiment was repeated at least three times.

### 4.8. Western Blot Analysis

Cells were extracted using the ice-cold Radio Immunoprecipitation Assay (RIPA) lysis buffer (Beyotime, P0013B). Protein concentration in the tissue and cell lysates were determined by BCA kits (Beyotime, P0010S). Then the protein samples were loaded, size-fractionated by SDS-PAGE gel, and transferred onto a Hybond chemiluminescence (ECL) transfer membrane (Amersham Pharmacia, Piscataway, NJ, USA). After blocking, the membranes were incubated with primary antibodies at 4 °C overnight. The primary antibodies for RECK, p53, Akt, phosphate-Akt, Bcl-2, Bax, matrix metalloproteinase 2 (MMP-2), *N*-Cadherin, and β-Actin were purchased from Cell Signaling Technology. The membranes were then incubated with the appropriate peroxidase conjugated secondary antibody (Santa Cruz, San Lucas, CA, USA). To visualize protein bands, ECL system (BIO-RAD, Hercules, CA, USA) was used.

### 4.9. qRT-PCR and RT-PCR

Total RNA from tissues and cells was isolated using Trizol reagent (Invitrogen, Grand Island, NY, USA) for both mRNA and miRNA analyses. Approximately 5 × 10^6^ cells were treated without (control) or with DHA for 48 h. miRNAs were isolated and purified using Trizol reagent, according to the manufacturer’s protocol. The concentration of total RNA was quantitated by measuring the absorbance at 260 nm (BioSpec-nano, Kyoto, Japan). Reverse transcription was performed using SuperScript III reverse transcriptase (Invitrogen). Quantitative real-time polymerase chain reaction (qRT-PCR) was performed with Platinum SYBR Green qPCR SuperMix-UDG (Invitrogen) and detected with the LightCycler 480 instrument (Roche, Basel, Switzerland). Fold change of miRNA and mRNA in the samples was determined using the comparative threshold cycle (*C*_t_) method (2^−ΔΔ*C*t^). The levels of U6 and glyceraldehyde-3-phosphate dehydrogenase (GADPH) were used to normalize miRNA and mRNA level, respectively. RECK and GADPH primers sequences used were synthesized by Sangon Biotech (Shanghai, China). Primers sequences used were synthesized by Sangon Biotech (Shanghai, China): hRECK: forward-5′-CATCACACAAACTGCCGAGAA-3′, reverse-5′-GGCGCAATAATTTTCCACTGCT-3′; hGADPH: forward-5′-CTCAAGGGCATCCTGGGCTAC-3′, reverse-5′-CAGCCCCAGCGTCA-AAGGT-3′. Primers of stem-loop method for miR-21-5p detection were synthesized by RiboBio (Guangzhou, China).

### 4.10. Transfection of Glioma Cells with miR-21 Inhibitor and miR-21 Mimic

MiR-21 was knocked down by transfection with a miRNA inhibitor, and it was over expressed by transfection with a miRNA mimic. MiR-21 inhibitor, mimic, and negative control were synthesized by RiboBio (Guangzhou, China). All of the oligonucleotides were transfected at a final concentration of 100 nM. Cells of 70%–80% confluence were transfected with oligonucleotides using RNAiMAX transfection reagents (Thermo Scientific, Waltham, MA, USA), according to the manufacturer’s recommendations. Transfection medium was replaced 6 h later.

### 4.11. Analysis of The Cancer Genome Atlas (TCGA) Expression Profile Data of GBM Tumor Tissues

Tertiary level expression data of GBM tumor tissues was downloaded from https://tcga-data.nci.nih.gov web site. For RECK expression levels, Affymetrix HT_HG U133A array data of 548 GBM tumor tissues was used for the analysis. Distribution of RECK expression levels was analyzed by frequency distribution. Detailed information used for the dataset was described in Table 2.

### 4.12. Statistical Analysis

Data were presented as mean ± SD (*n* = 3) except special indication. Student’s *t* test and one-way ANOVA were utilized for statistical analysis. A *χ*-square test was applied to determine the association of RECK levels with clinicopathological features. For all comparisons, *p* < 0.05 or fold change >2 was used as the criterion of significance.

## 5. Conclusions

**Figure A1:**
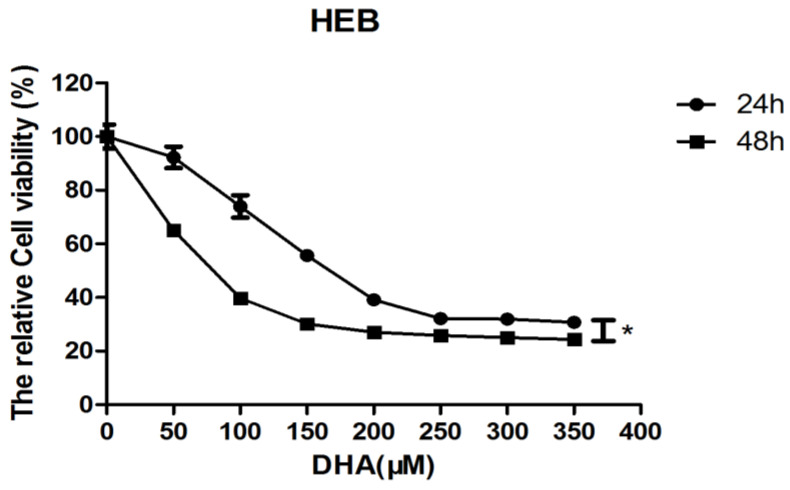
HEB was treated with a series of DHA concentrations (0–350 μM) for 24 or 48 h followed by MTT assay. Values were mean ± SD (*n* = 3). * *p* < 0.05 as compared with negative control cells.

In conclusion, our study provides a novel insight into the mechanisms of DHA in glioma cells and suggests that DHA is a potential effective agent for treating glioma. Furthermore, the neurotoxicity of dihydroartemisinin is one of its limits to the application in clinical practice (please see Figure A1 in the appendix); this indicates that there is a need to reduce the neurotoxicity and explore a proper usage for its application in clinical practice [7,14].

## Figures and Tables

**Table 1:** Correlation between RECK expression and clinicopathological features of glioma patients.

Clinical Characteristic	No. of Patients	RECK Expression Levels		*p*-Value
		High Expression (31)	Low Expression (57)	
Age (Year)				
<45	36	19	17	0.498
≥45	52	12	40	
Sex				
Male	53	16	37	0.655
Female	35	15	20	
Clinical Stage				
Low Grades I–II	45	25	20	0.021
High Grades III–IV	43	6	37	
Tumor Location				
Frontal	28	9	19	0.104
Parietal	5	2	3	
Occipital	6	0	6	
Temporal	22	10	12	
Others	27	10	17	

**Table 2:** RECK mRNA expression data from TCGA data.

RECK mRNA Expression Level	(4.0–4.5)	(4.5–5.0)	(5.0–5.5)	(5.5–6.0)	(6.0–6.5)	(6.5–7.0)	(7.0–7.5)
Samples	20	191	195	118	19	2	3

## Abbreviations

### Abbreviations

DHA	Dihydroartemisinin
MMP	Matrix metalloproteinase
RECK	Reversion-inducing-cysteine-rich protein with kazal motifs
