# Correction: Shao et al. AKT Axis, miR-21, and RECK Play Pivotal Roles in Dihydroartemisinin Killing Malignant Glioma Cells. *Int. J. Mol. Sci.* 2017, *18*, 350

**DOI:** 10.3390/ijms222312670

**Published:** 2021-11-24

**Authors:** Ying-Ying Shao, Tao-Lan Zhang, Lan-Xiang Wu, He-Cun Zou, Shuang Li, Jin Huang, Hong-Hao Zhou

**Affiliations:** 1 Institute of Life Sciences, Chongqing Medical University, 1 Yixueyuan Road, Yuzhong District, Chongqing 400016, China; yingying_shao1226@163.com (Y.-Y.S.); lxwu2008@126.com (L.-X.W.); zouhecun@outlook.com (H.-C.Z.); 2 Department of Clinical Pharmacology, Xiangya Hospital, Central South University, Changsha 410008, China; CSU_ZTL@163.com (T.-L.Z.); m15200923235@163.com (S.L.); huangjin879288@163.com (J.H.); 3 Hunan Key Laboratory of Pharmacogenetics, Institute of Clinical Pharmacology, Central South University, Changsha 410078, China

The authors wish to make the following corrections to this paper [1]: On page 4, the protein band of Actin in HS683 in Figure 2c was wrong. Thus, Figure 2c should be replaced with the following figure (Figure 2).

The authors apologize for any inconvenience caused and state that the scientific conclusions are unaffected.

## Figures and Tables

**Figure 2:**
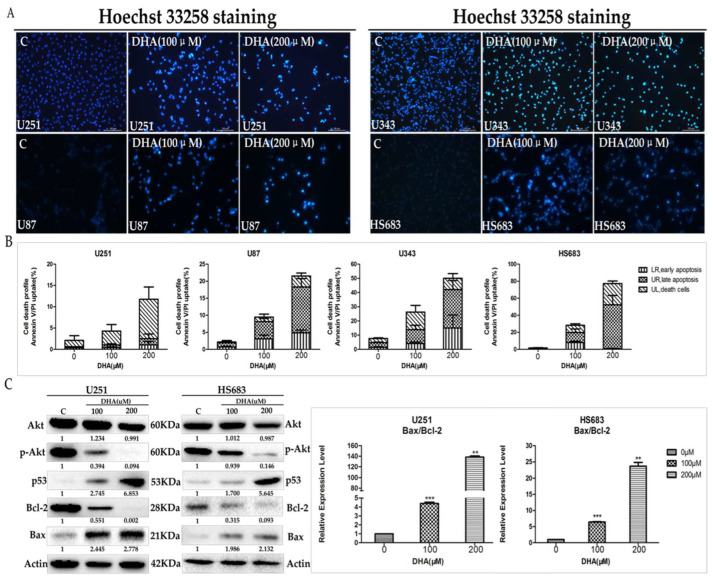
Effect of DHA on malignant glioma cells apoptosis by regulating AKT axis. The indicated cells were treated with 100 and 200 μM DHA for 48 h. (**A**) Cells stained with Hoechst 33258 were detected and calculated by fluorescent photomicrographs at 10×; (**B**) cells were labeled with Annexin V/Propidium Iodide (AnnexinV/PI) and detected by flow cytometry. Values were mean ± SD (*n* = 3); (**C**) the proteins associated with AKT/p53/Bcl-2/Bax axis in malignant glioma cells were determined by western blot analysis. The changes of Bax/Bcl-2 ratio were evaluated by western blot analysis. Values were mean ± SD (*n* = 3). ** *p* < 0.001, *** *p* < 0.0001 as compared with negative control cells.
